# Dysfunctions of Reward Motivation Adaptation in Patients With Schizophrenia

**DOI:** 10.1002/pchj.70056

**Published:** 2025-10-09

**Authors:** Yiming Pan, Hui Wang, Qi Zhou, Bingjie Huang, Chengcheng Pu, Simon S. Y. Lui, Jia Huang, Raymond C. K. Chan

**Affiliations:** ^1^ Neuropsychology and Applied Cognitive Neuroscience Laboratory, State Key Laboratory of Cognitive Science and Mental Health, Institute of Psychology, Chinese Academy of Sciences Beijing China; ^2^ Department of Psychology The University of Chinese Academy of Sciences Beijing China; ^3^ The Affiliated Brain Hospital of Nanjing Medical University Nanjing China; ^4^ Peking University Sixth Hospital Beijing China; ^5^ Department of Psychiatry School of Clinical Medicine, the University of Hong Kong Hong Kong China

**Keywords:** adaptation, effort‐reward imbalance, reward motivation, schizophrenia

## Abstract

Diminished reward motivation in the wanting or liking dimension constitutes one of the core dysfunctions in patients with schizophrenia (SCZ). However, it remains unclear whether patients with SCZ would dynamically adapt their wanting or liking towards reward in response to a favourable effort‐reward ratio and whether such adaptation correlates with their clinical symptoms or functional outcome. In this study, thirty patients with SCZ and 30 healthy controls (HC) were recruited to complete the reward motivation adaptation task (RMAT) based on mental arithmetic effort and manipulating effort‐reward ratios. Clinical symptoms were assessed in the clinical group while pleasure experience and social functioning were assessed in all participants. We found that patients with SCZ exhibited less reward wanting and liking than HC in “effort = reward” and “effort < reward” conditions. Neither reward wanting nor liking in patients with SCZ adapted with effort‐reward ratio as indicated by significantly smaller coefficients (βwanting and βliking) compared with HCs. Besides, SCZ patients' adaptation ability was positively correlated with social functioning in daily life. In conclusion, this study indicates that patients with SCZ not only exhibited reduced reward motivation in favourable conditions but also dysfunctions of reward motivation adaptation, and such deficits could explain poor functional outcome.

## 1 Introduction

The willingness to expend effort for potential pleasurable outcomes is known as reward motivation, which plays an important role in humans' goal‐directed behaviour (Waugh and Gotlib 2008). Reward motivation could vary with the change in reward magnitude and probability. For instance, healthy people may become less motivated in non‐rewarding conditions but highly motivated if the conditions change and become rewarding (Treadway et al. 2009; Yan et al. 2023). The effort‐reward imbalance (ERI), which pertains to the perception of expending considerable effort yet receiving disproportionately little reward (Siegrist 2002), may provide a plausible framework to examine the reward motivation adaptation (Yan et al. 2023). Preliminary findings showed that higher ERI could predict diminished reward motivation in both work and school environments (Omansky et al. 2016; Pan et al. 2023).

Diminished reward motivation refers to reduced willingness to pursue high reward at the expense of making high effort, contributing to avolition or anhedonia, which are core negative symptoms in patients with schizophrenia (SCZ) (Llerena et al. 2013; Wolf et al. 2014; Wang et al. 2015). Previous studies on altered reward motivation in SCZ patients have predominantly employed *static* and *self‐paced* behavioural paradigms, for example, effort‐based decision‐making paradigms (Hartmann‐Riemer et al. 2018). Inherently, these paradigms have low ecological validity and may not capture the real‐time cost–benefit computation due to uncontrolled stressful conditions. However, in non‐decision ERI situations, patients usually feel obliged to exert effort regardless of the effort cost level. It is understandable that ERI situations would diminish SCZ patients' reward motivation because such situations may be perceived as “uncontrollable” and “stressful”. Uncontrollable stressful conditions could disrupt the mesocortical and mesolimbic dopamine transmission and reduce reward responsiveness, resulting in motivation deficits and anhedonia (Pizzagalli 2014). Since reward motivation dysfunctions are associated with negative symptoms in SCZ patients, it is necessary to evaluate the dysfunctions of reward motivation adaptation with varying stress situations. A previous experience sampling study found that individuals with clinical high risk for developing psychosis and patients with first‐episode psychosis showed higher levels of anhedonia than controls, but the impact of stress on the levels of anhedonia appeared to be similar in the three groups (Gerritsen et al. 2019). Therefore, the impact of stressful ERI on SCZ patients' reward motivation adaptation needs to be further explored.

Despite the paucity of evidence on reward motivation adaptation in SCZ patients, previous studies using subclinical populations along the SCZ spectrum suggested that people with negative schizotypal traits may have similar but attenuated avolition or anhedonia symptoms as SCZ patients (Kwapil et al. 2013; Barrantes‐Vidal et al. 2015) and tended to perceive higher ERI (Yan et al. 2021). The novel Reward Motivation Adaptation Task (RMAT) (Yan et al. 2023) measures reward motivation *adaptation* by evaluating both “wanting” and “liking” components (Berridge 2003) under *dynamically changing* effort‐reward ratio conditions. “Wanting” (i.e., willingness to exert effort for reward) involves the mesolimbic dopamine system and activates one's behaviour to pursue reward. “Liking” (i.e., consummatory pleasure after receiving actual rewards) is related to the mesolimbic opioid system and leads to one's enjoyment of rewards (Berridge 2003; Pessiglione et al. 2007; Berridge and Robinson 1998). College students with negative schizotypal traits reported generalized impairments of “wanting”, “liking” and reward motivation adaptation (Yan et al. 2023), but SCZ patients exhibited deficits only in ‘wanting’ but not ‘liking’ (Catalano et al. 2022). Whether SCZ patients have distinct dysfunction patterns during reward adaptation remained unclear.

It is important to clarify the dysfunction patterns of both “wanting” and “liking” in different effort‐reward (E‐R) conditions in SCZ patients, for unveiling the cognitive mechanisms of motivation deficits based on the anticipatory and consummatory theoretical perspective (Fervaha et al. 2014; Kring and Barch 2014). Furthermore, the dynamic adaptation affecting ‘wanting’ and ‘liking’ has high ecological validity to SCZ patients' daily life. Previous meta‐analytic evidence revealed that SCZ patients who showed higher levels of resilience exhibited better and more improved social functioning (Wambua et al. 2020). Thus, it could be presumed that if patients' motivation could “rebound” in non‐ERI situations, their functional outcomes may be less likely affected by the stressful ERI situations.

The current study aimed to explore whether SCZ patients would be able to adapt their reward motivation according to various effort–reward conditions. We also aimed to clarify whether reward motivation adaptation in SCZ patients would be correlated with negative symptoms and social functioning. Based on prior studies in subclinical populations with high levels of schizotypal traits, we hypothesized that (1) SCZ patients would not have enhanced reward motivation when the effort–reward condition becomes favorable, and (2) the reward motivation adaptation dysfunction would be correlated with negative symptoms and social functioning in SCZ patients.

## 2 Methods

### 2.1 Participants

G‐power software was used to calculate the required sample size to detect within‐between interaction effect (two groups, three repeated measures) for the reward motivation behavioural task, with estimated medium effect size *f* = 0.250, *α* = 0.050, power (1‐β) = 0.950. The total required sample size was 44. We therefore recruited 30 SCZ patients and 30 controls in this cross‐sectional study.

First, 30 outpatients with SCZ from Beijing Sixth Hospital, Beijing, China in 2022. Inclusion criteria of SCZ were (1) meeting the diagnostic criteria of SCZ according to DSM‐5, and duration of SCZ ≤ 10 years; (2) 18–55 years old; (3) education ≥ 9 years, being able to read and comprehend task demands and cooperate with researchers to complete the task; (4) estimated IQ ≥ 70; (5) right‐handed. Exclusion criteria of SCZ were (1) substance dependence or abuse; (2) history of brain injury or neurological disorders; (3) used physical therapy like transcranial magnetic stimulation (TMS) or electroconvulsive therapy (ECT) in the past 12 weeks at the time of recruitment; (4) comorbidity with other psychiatric disorders, like bipolar disorder or major depressive disorder.

Then, 30 healthy controls (HC) matched with SCZ participants in age and gender were recruited from universities and communities around the hospital by advertisements and posters. The inclusion and exclusion criteria of HC were the same as those for SCZ participants, apart from the absence of any personal and family history of psychiatric disorders.

### 2.2 Measures

#### 2.2.1 IQ Measures

We estimated IQ using the short form of the Wechsler Adult Intelligence Scale‐Revised (WAIS‐R)—Chinese version (Gong 1992), which consists of four domains: general knowledge, arithmetic, similarities, and digit span.

#### 2.2.2 Clinical Assessments

All clinical assessments for SCZ participants were conducted by experienced clinical psychiatrists in Beijing Sixth Hospital. The Positive and Negative Syndrome Scale (PANSS) (Kay et al. 1987; He and Zhang 2000) was used to assess positive, negative, and general symptoms for patients with SCZ; higher scores of each dimension reveal severer symptoms. The Clinical Assessment Interview for Negative Symptoms (CAINS) (Kring et al. 2013; Chan et al. 2015), a semi‐structured clinical interview, was used to assess patients' motivation and pleasure (MAP) and expression (EXP) factors, and higher scores of each factor represent more impairments.

The Calgary Depression Scale for Schizophrenia (CDSS) (Addington et al. 1990; Xiao et al. 2009) was used to assess accompanying depressive symptoms for patients with SCZ. The Simpson‐Angus Scale (SAS) was used to assess extrapyramidal side effects elicited by antipsychotic medications, including rigidity, blink, tremor, and salivation (Simpson and Angus 1970). Higher scores of these scales reflect more severe corresponding symptoms.

#### 2.2.3 Self‐Report Questionnaires

The Temporal Experience of Pleasure Scale (TEPS) was used to measure individuals' ability to experience both anticipatory and consummatory pleasure (Gard et al. 2006; Chan, Shi, et al. 2012). Higher scores in anticipatory or consummatory dimensions revealed a better ability to experience pleasure.

The First Episode Social Functioning Scale (FESFS) was used to measure social functioning for first‐episode psychosis (Lecomte et al. 2014, 2005; Wang et al. 2013), including living skills, interpersonal, social activities, intimacy, friends, family, work, and school dimensions. Higher scores for each dimension mean better corresponding social functioning.

The Chapman Social Anhedonia Scale (CSAS) (Chan, Wang, et al. 2012; Chapman et al. 1976) was used to measure the degree of impairment in the ability to experience social pleasure. Higher total scores mean more severe anhedonia symptoms.

### 2.3 Behavioural Task: Reward Motivation Adaptation Task—Patient Version

Based on the effort‐reward imbalance model, this paradigm was a mental arithmetic task adapted from the reward motivation adaptation task (RMAT) (Yan et al. 2023), written by PsychoPy (v2021.2.3). Independent variables included three effort‐reward conditions: “effort = reward” (average, A) vs. “effort > reward” (bottom, B) vs. “effort < reward” (top, T). Effort‐reward relationships were manipulated by the ratio of actual reward feedback and expected reward range; a ratio < 1 indicated “effort > reward”, ratio = 1 indicated “effort = reward”, and a ratio > 1 indicated “effort < reward”. Dependent variables were two indicators of reward motivation: “wanting” and “liking”. Given that patients with SCZ had weaker cognitive functioning than healthy controls, we adjusted the task design based on the original RMAT: reduced four trials to 16 trials for each condition in the current task (a total of 48 trials), and lengthened the duration of each arithmetic item from 1 s to 1.5 s to ensure patients could finish the task. The paradigm used an ABATATAB block design, each A block having 5 trials and each B and T block having 10 trials.

**FIGURE 1:**
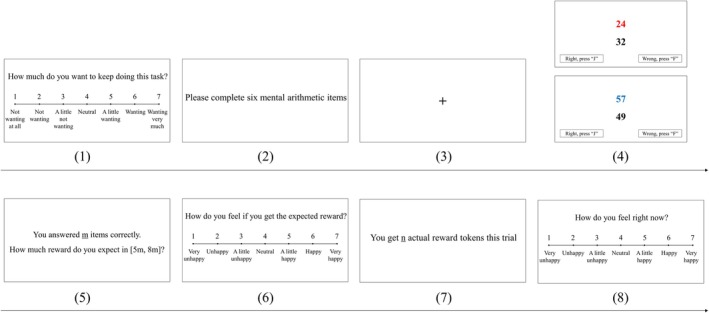
Schematic diagram of the reward motivation adaptation task (RMAT) – patient version.

Figure 1 illustrates the running of the experimental task. When a trial began (except the first trial), participants were required to rate whether they would like to continue this task (1–7 scores, 1: not at all, 7: very much), as the indicator of “reward wanting”. Then they were required to finish six mental arithmetic items. Red numbers and blue numbers were randomly displayed in the sequence on the screen, and participants needed to perform +8 and −8 calculations respectively and identify whether the outcome represented by the black number was true (press “J” key) or false (press “F” key) within 1.5 s. If they had no response or reaction time ≤ 200 ms for more than 2 of 6 items, they had to restart this trial. After finishing the six items, the number of items answered correctly would be displayed on the screen, and participants needed to input an expected reward ranging from the product of 5 and the number of correct items to the product of 8 and the number of correct items, and evaluate their anticipatory pleasure experience (1–7 scores, 1: *very unhappy*, 7: *very happy*). Lastly, participants received feedback on actual rewards in this trial and evaluated their consummatory pleasure experience (1–7 scores, 1: *very unhappy*, 7: *very happy*), as the indicator of “reward liking”.

### 2.4 Procedure

All assessments were conducted face‐to‐face, in a quiet room, under the guidance of trained researchers. After listening to the procedure and requirements of the experiment, participants provided written informed consent. Researchers then gathered basic demographic information, including age, gender, years of education, employment status, marital status, current living companions, self‐care ability, lifestyle habits (smoking and alcohol consumption), and medical histories. Additionally, they estimated clinical participants' duration of illness and treatment status, and completed the clinical rating scales. Lastly, participants completed the behavioral task and self‐reported questionnaires posted online (https://www.wjx.cn/vm/mFwRTdV.aspx). Participants received monetary compensation after completion of the assessments.

### 2.5 Data Analysis

Data collected in the study was analyzed using SPSS 22.0. The statistical significance level was set at *p* < 0.050 (two‐tailed). For the behavioural task, we calculated the mean value of “wanting” and “liking” in three conditions (effort = reward, effort > reward, effort < reward) respectively as the indicator of reward motivation. We calculated the mean accuracy (ACC) and mean reaction time (RT) for six times of mental arithmetic in each trial and defined “effort efficiency” as mean ACC/mean RT, to control possible discrepancies between different trials and participants. Then we conducted three repeated ANOVA analyses with “wanting”, “liking” and effort efficiency as the dependent variables respectively, and entering the main effects of “effort‐reward relationship” and “group” and their interaction as the independent variables to examine reward motivation patterns of SCZ patients in varying effort‐reward conditions. We conducted the Greenhouse–Geisser correction with adjusted degrees of freedom when the sphericity assumption in Mauchly's test could not be met.

Reward motivation adaptation was calculated by constructing two regression models with “wanting” and “liking” as the dependent variables respectively, reward‐effort ratio (actual reward feedback/the expected reward input by participants) as the independent variable, and trial number as a covariate to eliminate effects of fatigue and practice. Regression coefficients *β*_*wanting*_ and *β*_*liking*_ from the two models were defined as the indicators of reward motivation adaptation to investigate the flexibility of adapting reward motivation for SCZ patients. We conducted Pearson correlation analysis between the regression coefficients and the ratings on self‐reported questionnaires to unveil the potential clinical value of adaptation coefficients, and Chi‐square tests and independent‐sample t tests were performed to compare the differences in demographic information and self‐reported questionnaires between SCZ patients and HC.

## 3 Results

### 3.1 Demographic Information and Clinical Characteristics

Table 1 shows the characteristics of the sample. Thirty SCZ patients (14 female; 29.70 ± 6.62 years) and 30 HCs (15 female; 27.77 ± 6.37 years) finished the experiment. SCZ patients showed comparable age, gender ratio, education years, and IQ estimates with HCs (*p*s > 0.050), but were more often unemployed (Fisher exact test, *p* < 0.001) and living with parents (Fisher exact test, *p* < 0.001) than HCs. On average, our SCZ participants had been admitted once to psychiatric hospitals (1.03 ± 1.93 times) and had one psychotic relapse (1.28 ± 1.79 times). They also showed considerable psychopathological symptoms (PANSS total scores: 45.97 ± 8.01), but little depressive symptoms (CDSS: 0.83 ± 1.15) and parkinsonism side effects (SAS: 0.40 ± 0.62).

**TABLE 1:** Basic demographic information and clinical characteristics.

	SCZ (*n* = 30)	HC (*n* = 30)	*t*/*χ* ^2^(df)	*p*
	*Mean* (SD)	*Mean* (SD)		
Age (years)	29.70 (6.62); 21–45	27.77 (6.37); 20–42	1.153 (58)	0.254
Gender (male/female)	16/14	15/15	0.067 (1)	0.796
Education years	14.93 (2.50)	15.17 (2.71)	−0.347 (58)	0.730
IQ estimates	107.27 (14.21)	113.73 (14.87)	−1.722 (58)	0.09
Employment status^a^				
Unemployed	14 (46.7%)	1 (3.3%)	19.066	< 0.001
Employed	14 (46.7%)	29 (96.7%)		
On Sick Leave/Leave of Absence	2 (6.7%)	0		
Marital Status^a^				
Single	21 (70.0%)	18 (60.0%)	3.011	0.427
Married	6 (20.0%)	11 (36.7%)		
Separation	1 (3.3%)	0		
Divorced	2 (6.7%)	1 (3.3%)		
Current cohabitants^a^				
Living alone	6 (20.0%)	15 (50%)	19.144	< 0.001
Spouse	4 (13.3%)	7 (23.3%)		
Parents	17 (56.7%)	4 (13.3%)		
Extended relatives	0	4 (13.3%)		
prefer not to disclose	3 (10.0%)	0		
Self‐care ability^a^				
Independent living	26 (86.7%)	30 (100%)		
Requiring assistance help in some complex activities	4 (13.3%)	0	—	0.112
Smoking^a^				
Never	27 (90.0%)	27 (90.0%)	5.172	0.117
≤ 10 cigarettes/day	0	3 (10.0%)		
≥ 21 cigarettes/day	1 (3.3%)	0		
prefer not to disclose	2 (6.7%)	0		
Alcohols drinking^a^				
Never	28 (93.3%)	30 (100%)	—	0.472
Occasional	2 (6.7%)	0		
Hospitalization (times)	1.03 (1.93)			
From illness occurrence to first treatment (months)^b^	9.59 (12.16)			
Recurrence (times)	1.28 (1.79)			
Antipsychotics medication (Chlorpromazine equivalence; mg/day)^c^	408.49 (309.19)			
PANSS				
Positive symptoms	9.30 (1.84)			
Negative symptoms	14.57 (4.08)			
General symptoms	22.10 (3.86)			
Total score	45.97 (8.01)			
CAINS				
MAP	17.17 (6.51)			
EXP^c^	5.32 (2.92)			
Total score^d^	21.79 (8.08)			
CDSS	0.83 (1.15)			
SAS	0.40 (0.62)			

Abbreviations: CAINS, The clinical assessment interview for negative symptoms; CDSS, Calgary depression scale for schizophrenia; EXP, Expression factor subscale; MAP, motivation and pleasure factor subscale; PANSS, The positive and negative SYNDROME Scale; SAS, Simpson‐angus scale.

^a^ Fisher's exact test was used because the expected count was less than 5 in at least two cells.

^b^ Valid sample size of SCZ = 29.

^c^ Valid sample size of SCZ = 25.

^d^ Valid sample size of SCZ = 28.

### 3.2 Reward Motivation Adaptation Task (RMAT) Performance

We explored the influence of different effort‐reward conditions, groups, and their interaction on “wanting”, “liking” and “effort efficiency” by using multivariate repeated ANOVA. No covariate was included in ANOVA, as there was no group difference in age, gender ratio, education years, and IQ estimates.

As shown in Table 2, effort‐reward conditions had significant main effects on both “wanting” and “liking”, “wanting”: *F*(1.332, 77.257) = 21.555, *p* < 0.001, *η*_*p*_^2^ = 0.271; “liking”: *F*(1.188, 68.926) = 82.270, *p* < 0.001, *η*_*p*_^2^ = 0.587, which indicated the validity of the effort‐reward condition, but no significant main effects on effort efficiency, *F*(1.848, 107.157) = 2.669, *p* = 0.078, *η*_*p*_^2^ = 0.044, indicating that participants' degree of effort expenditure in three conditions was similar.

**TABLE 2:** Results of repeated ANOVA in behavioural task.

Independent variables	Dependent variables	*F*(df*1*, df*2*)	*p*	*η* ^2^_*p*_
Effort‐reward relationship	Wanting	21.555 (1.332, 77.257)	< 0.001	0.271
	Liking	82.270 (1.188, 68.926)	< 0.001	0.587
	Effort efficiency	2.669 (1.848, 107.157)	0.078	0.044
Group	Wanting	3.135 (1, 58)	0.082	0.051
	Liking	4.559 (1, 58)	0.037	0.073
	Effort efficiency	0.067 (1, 58)	0.796	0.001
Effort‐reward relationship * Group	Wanting	9.190 (1.332, 77.257)	0.001	0.137
	Liking	15.172 (1.188, 68.926)	< 0.001	0.207
	Effort efficiency	0.798 (1.848, 107.157)	0.444	0.014

The Group main effect indicated that SCZ patients had significantly lower “liking” than HC (*F*(1, 58) = 4.559, *p* = 0.037, *η*_*p*_^2^ = 0.073), and marginally lower “wanting” than HC (*F*(1, 58) = 3.135, *p* = 0.082, *η*_*p*_^2^ = 0.051), implicating diminished pleasure for obtained reward and a tendency for reduced motivation for pursuing reward in SCZ patients. There was no significant difference in effort efficiency for SCZ and HC (*F*(1, 58) = 0.067, *p* = 0.796, *η*_*p*_^2^ = 0.001), indicating that the two groups had a similar degree of effort expenditure.

**FIGURE 2:**
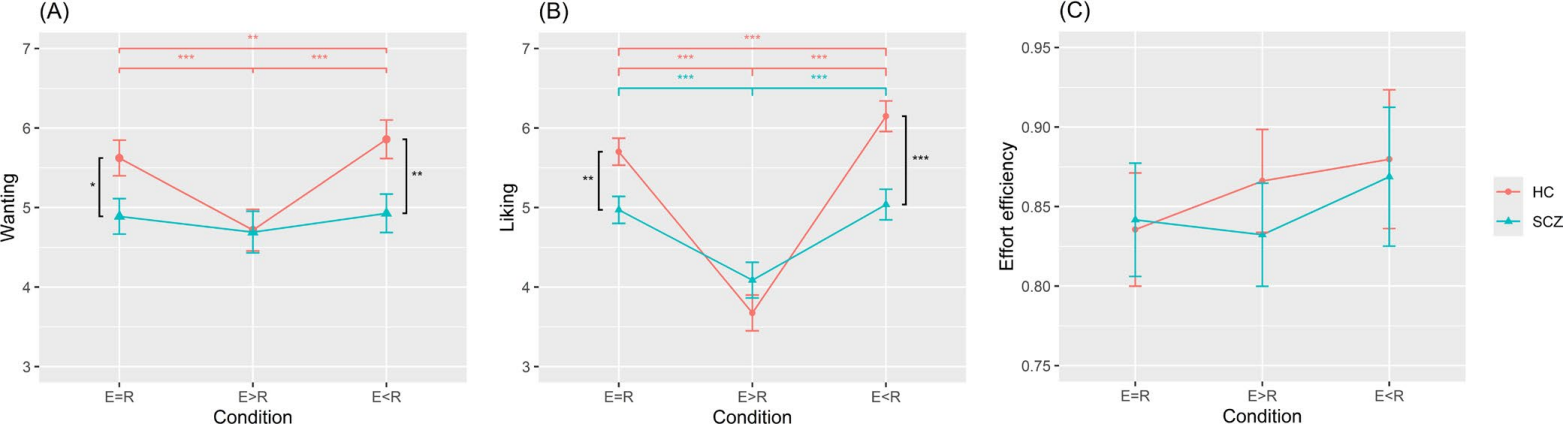
Line charts of (A) wanting, (B) liking, and (C) effort efficiency in three effort‐reward conditions for two groups. The error bars represent standard error in MANOVA, and the black, red and green significance label respectively represent comparisons between SCZ and HC in the same condition, among SCZ's three conditions, and among HC's three conditions. E = *R*: Effort = reward. E>*R*: Effort > reward. E<*R*: Effort < reward. *P < 0.050, **P < 0.010, ***P < 0.001.

The interaction between the effort‐reward relationship and group was significant on both “wanting” and “liking”, “wanting”: *F*(1.332, 77.257) = 9.190, *p* = 0.001, *η*_*p*_^2^ = 0.137; “liking”: *F*(1.188, 68.926) = 15.172, *p* < 0.001, *η*_*p*_^2^ = 0.207, but there was no significant main effect on effort efficiency, *F*(1.848, 107.157) = 0.798, *p* = 0.444, *η*_*p*_^2^ = 0.014, suggesting that the reward motivation of the two groups showed different adaptation patterns when effort‐reward varied, but a similar degree of effort expenditure. Simple effects analysis further found that “wanting” and “liking” of patients with SCZ were both significantly lower than HC in “effort = reward balance” and “effort < reward imbalance” conditions, but were not significant in the “effort > reward imbalance” condition (Table 3 and Figure 2).

**TABLE 3:** Simple effects analysis of effort‐reward x group interaction.

Effort‐reward condition	Dependent variables	SCZ (*n* = 30)	HC (*n* = 30)	*F*(1,58)	*p*	*η* ^2^_*p*_
		Mean (SD)	Mean (SD)			
Effort = reward	Wanting	4.89 (0.22)	5.62 (0.22)	5.378	0.024	0.085
	Liking	4.97 (0.17)	5.7 (0.17)	9.282	0.003	0.138
	Effort efficiency	0.84 (0.04)	0.84 (0.04)	0.015	0.904	< 0.001
Effort > reward	Wanting	4.69 (0.26)	4.72 (0.26)	0.005	0.945	< 0.001
	Liking	4.09 (0.22)	3.68 (0.22)	1.689	0.199	0.028
	Effort efficiency	0.83 (0.03)	0.87 (0.03)	0.545	0.463	0.009
Effort < reward	Wanting	4.93 (0.24)	5.86 (0.24)	7.414	0.009	0.113
	Liking	5.04 (0.19)	6.15 (0.19)	16.702	< 0.001	0.224
	Effort efficiency	0.87 (0.04)	0.88 (0.04)	0.032	0.859	0.001

### 3.3 Self‐Report Questionnaires and Reward Motivation Adaptation Coefficients (β_wanting_ and β_liking_)

As shown in Table 4, the *β*_*wanting*_ and *β*_*liking*_, which represented the extent of reward motivation adaptation in the behavioural task, were both lower in SCZ patients than in HC (*β*_*wanting*_: *t*(54.275) = −2.889, *p* = 0.005; *β*_*liking*_: *t*(58) = −3.448, *p* = 0.001). Among the self‐report questionnaires, the SCZ group's ratings on CSAS, TEPS consummatory pleasure subscale, and total scores were significantly lower than HC (CSAS: *t*(58) = −2.701, *p* = 0.009; TEPS consummatory pleasure: *t*(58) = −2.439, *p* = 0.018; TEPS total score: *t*(58) = −2.137, *p* = 0.037). Regarding social functioning, the social activities, school dimension, and total scores of FESFS were significantly lower in the SCZ group than in HC (social activities: *t*(26.351) = −1.937, *p* = 0.035; school: *t*(16.887) = −2.754, *p* = 0.014; total scores: *t*(26.666) = −2.469, *p* = 0.020).

**TABLE 4:** Descriptive statistics of reward motivation adaptation and scale scores.

	SCZ	HC	*t* (df)	*p*
	Mean (SD)	Mean (SD)		
*β*_wanting_	0.18 (0.23)	0.38 (0.30)	−2.889 (54.275)	0.005
*β*_liking_	0.27 (0.45)	0.64 (0.33)	−3.448 (58)	0.001
TEPS				
Anticipatory pleasure	33.77 (8.81)	37.30 (8.38)	−1.592 (58)	0.117
Consummatory pleasure	40.67 (10.1)	46.87 (9.59)	−2.439 (58)	0.018
Total scores	78.60 (17.95)	88.53 (18.05)	−2.137 (58)	0.037
CSAS	12.67 (7.18)	8.13 (5.73)	2.701 (58)	0.009
FESFS^a^				
Living skills	15.55 (2.21)	16.3 (2.39)	−1.118 (48)	0.269
Interpersonal	16.45 (3.58)	17.1 (3.03)	−0.691 (48)	0.493
Social activities	10.85 (2.92)	12.23 (1.57)	−1.937 (26.351)	0.035
Intimacy	12.25 (2.65)	12.93 (1.57)	−1.037 (48)	0.309
Friends	8.1 (2.38)	9.23 (1.33)	−1.936 (26.967)	0.063
Family	14.85 (1.93)	14.93 (2.61)	−0.122 (48)	0.903
Work	21.7 (3.33)	20.95 (2.31)	0.723 (28)	0.476
School	20.2 (0.84)	22.5 (2.79)	−2.754 (16.887)	0.014
Total Scores	93.95 (21.91)	107.20 (12.00)	−2.469 (26.666)	0.020

*Note*: Work dimension: NSCZ = 10, NHC = 20. School dimension: NSCZ = 5, NHC = 15. Other dimensions: NSCZ = 20, NHC = 30.

Abbreviations: CSAS, Chapman Social Anhedonia Scale; FESFS, The First Episode Social Functioning Scale. TEPS, Temporal Experience of Pleasure Scale.

^a^ 20 SCZ patients filled in the FESFS questionnaire.

**FIGURE 3:**
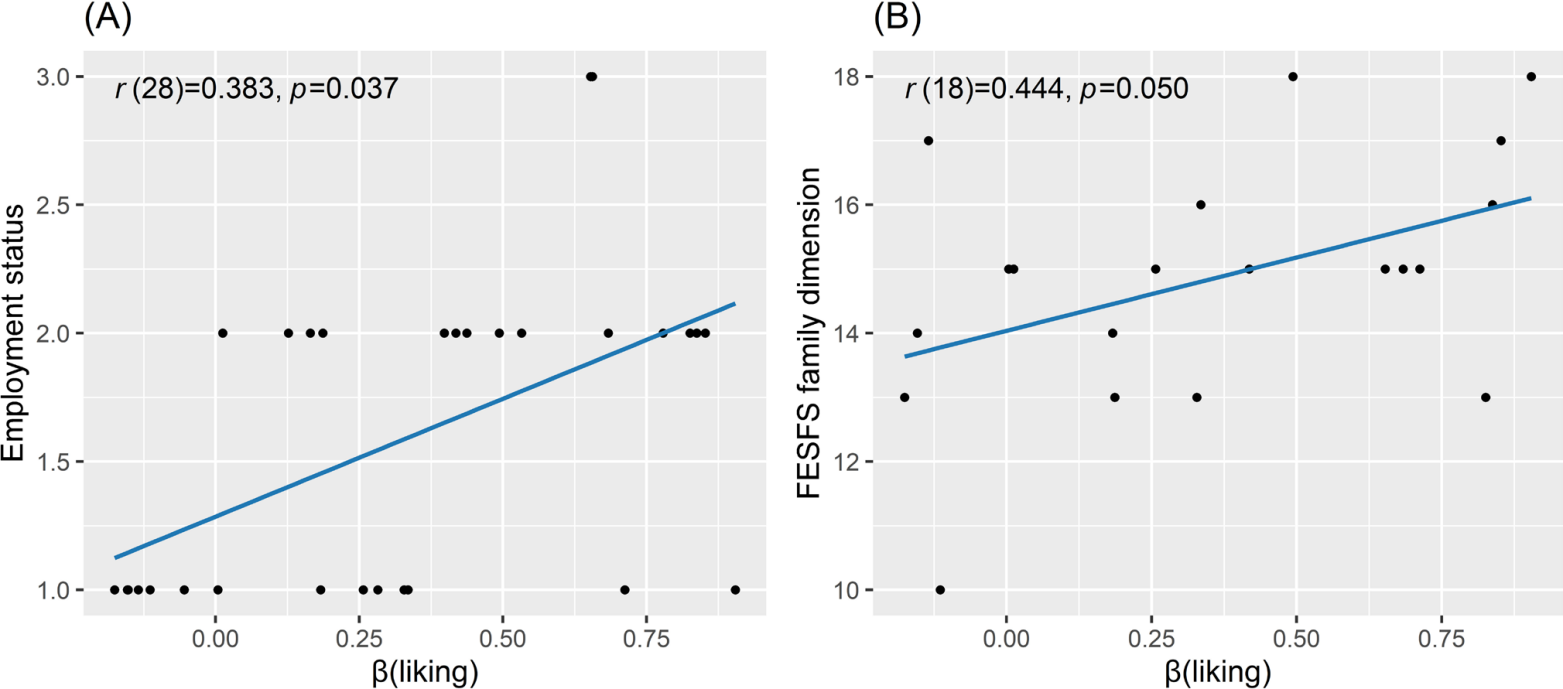
Scatter plots of correlation between *β*_*liking*_ and (A) employment status (B) family dimension in FESFS in SCZ patients.

Regarding the relationship between reward motivation and symptoms, the two reward motivation adaptation coefficients (*β*_*wanting*_ and *β*_*liking*_) were significantly correlated in both the SCZ and HC groups (SCZ: *r*(28) = 0.386, *p* = 0.035; HC: *r*(28) = 0.500, *p* = 0.005). In SCZ patients, there was no significant correlation between adaptation coefficients and negative symptoms (*p*s > 0.050). *β*_*liking*_ showed a significant positive correlation with employment status (1 = Unemployed/On Sick Leave/Leave of Absence, 2 = Employed) (*r*(28) = 0.383, *p* = 0.037) and family dimension in FESFS (*r*(18) = 0.444, *p* = 0.050) (Figure 3). No significant difference between antipsychotic equivalents and *β* coefficients in SCZ patients could be found (*p*s > 0.050), indicating that patients' adaptation pattern was not influenced by medication use.

In HC, we did not find any correlation between *β*_*wanting*_ and any self‐reported questionnaires (*p*s > 0.050), and *β*_*liking*_ showed a significant positive correlation with the friends dimension in FESFS (*r*(28) = 0.472, *p* = 0.008), revealing that interaction with friends and support from friends would show a greater impact on the adaptation of liking reward.

## 4 Discussion

In line with our hypothesis, SCZ patients showed dysfunctions in reward motivation adaptation in both wanting and liking components, and the attenuated adaptation in SCZ patients was correlated with poor social functioning in daily life. Our findings concurred with previous results that SCZ patients had difficulties in initiating goal‐directed behavior in favorable conditions such as high‐reward or positive‐event conditions, while their performances did not differ from the controls in low‐reward or negative‐event conditions (Huang et al. 2016; Campellone et al. 2018). We provided the inaugural evidence of impaired reward motivation adaptation in SCZ patients when they were facing dynamically uncontrollable situations. Furthermore, similar to the performances of individuals with negative schizotypal traits (Yan et al. 2023), SCZ patients could not enhance their reward motivation when the situation gets better, suggesting that such dysfunctions of reward motivation adaptation may exist widely in the schizophrenia spectrum populations.

Specially, we have calculated coefficients *β* (*β*_wanting_ and *β*_liking_) for each individual to measure the adaptation ability of reward motivation. Higher *β* demonstrated that an individual could modulate wanting and liking towards reward, according to the favourability of effort‐reward ratios in each trial (a higher ratio of reward/effort means more favourable). This trial‐by‐trial analysis would exclude the influence of fatigue and practice effects by setting trial number as a covariate, but the previous study only compared the levels of reward wanting and liking between conditions (Yan et al. 2023). The calculations of *β*_wanting_ and *β*_liking_ may provide effective evaluations of reward motivation adaptation.

Failures in reward motivation adaptation may lead to less reward pursuit when the situation becomes better, which may pose challenges to rehabilitation training and affect SCZ patients' daily life. A plausible explanation of the diminished reward motivation adaptation in SCZ patients may be related to the childhood antecendents involving social maladjustments (Done et al. 1994), or impaired self‐regulation for goal‐directed behaviour (Orellana and Slachevsky 2013). Moreover, it is also plausible that SCZ patients would tend to overestimate effort cost and underestimate reward value, which can impede the optimal choice and action (Treadway et al. 2015). Furthermore, dysfunctions of reward motivation in SCZ patients in daily life (Messinger et al. 2011; Rector et al. 2005) may be due to the lack of positive response to external condition (Huang et al. 2016; Park et al. 2017). Hence, SCZ patients would be more likely exposed to stress environment in the long term (Walker and Diforio 1997; Cougnard et al. 2007). Experience sampling method (Visser et al. 2020) may be helpful to clarify how altered reward motivation adaptation could impede the rehabilitation of SCZ patients in real‐life.

Moreover, diminished reward motivation adaptation in SCZ patients may reflect a developmental continuity. Specifically, altered dynamic modulation of reward valuation in adulthood can be traced back to patients' pre‐morbid early‐life social maladjustments (Done et al. 1994) and executive dysregulation persisting into adulthood (Orellana and Slachevsky 2013). Critically, SCZ patients with prominent negative symptoms exhibited diminished effort expenditure despite escalating reward incentives, indicating a core deficit in reward motivation (Wang et al. 2015). This impairment is rooted in disrupted effort‐reward computation, a cognitive mechanism directly linked to negative symptom psychopathology. By implementing the novel RAMT paradigm, our adaptation framework transcends prior static models of motivational deficits. Crucially, we demonstrated that SCZ involves not merely aberrant effort allocation, but a fundamental failure in adaptively computing effort‐reward tradeoffs, which may serve as the psychopathological mechanism underlying avolition. A meta‐analysis found strong relationships between motivation and negative symptoms as measured by second‐generation negative symptom measures (Luther et al. 2019). Nevertheless, our findings failed to suggest that reward motivation adaptation would be correlated with the pleasure experience factor of the CAINS. One possible explanation could be that the pleasure experience factor of the CAINS taps into the retrospective summary about patients' past experience, and may not effectively assess patients' current, dynamic changes in reward motivation (Blanchard et al. 2020). Alternatively, consistent with previous research implicating no effect of antipsychotic medications on motivation (Fervaha et al. 2015), we did not find any correlation between medication dosage and motivation deficits in SCZ patients, suggesting that these deficits of reward motivation adaptation persist independently of medication effects.

We also found that higher adaptation of reward liking in SCZ patients was correlated with a greater possibility of finding a job for the return to society and also a closer relationship with family. In previous studies, extrinsic motivation was positively related to interpersonal functioning, such as interpersonal behaviour and employment (Uchino et al. 2021), and motivation and pleasure deficits predicted poor functional outcomes for SCZ patients (Hu et al. 2022; Lui et al. 2023). Our results have expanded these prior findings by showing that the adaptation of reward motivation in response to favourable environmental conditions would also serve an important role in the rehabilitation of social functioning for SCZ patients.

Several limitations in our study should be borne in mind. First, although we had modified the RMAT paradigm to make it easier for clinical patients, the mental effort in this task may still be difficult for SCZ patients, and they may become unwilling to exert effort cost even though the effort is low and the reward value is high. Second, we did not examine the brain functioning of SCZ patients. Future studies may further examine the neural correlates of the reward motivation adaptation to understand the neural mechanisms for the dysfunction of reward motivation adaptation.

## 5 Conclusion

This study indicated that SCZ patients exhibited dysfunctions of reward motivation adaptation, which were associated with their negative symptoms and poor social functioning. Our work illustrated a novel attempt to understand the cognitive mechanism underlying the dynamic adaptation of reward motivation in schizophrenia, complementing our current knowledge on the static reward motivation in SCZ patients. This approach may shed new insights on the cognitive mechanisms underlying amotivation and anhedonia in SCZ patients, and explore further the important role of reward motivation adaptation in affecting clinical symptoms and functional outcomes. Future studies could further explore the neurophysiological mechanisms underlying this impaired adaptation ability in populations with SCZ spectrum disorders.

## Ethics Statement

This study was approved by the Ethics Committee of the Institute of Psychology, the Chinese Academy of Sciences (Protocol number: H20041) and the Ethics Committee of Peking University Sixth Hospital (Protocol number: 202234) as a collaborative project.

## Conflicts of Interest

The authors declare no conflicts of interest.

## Data Availability

The data that support the findings of this study are available from the corresponding author upon reasonable request.
